# Out-of-Hospital Cardiac Arrest –Optimal Management

**DOI:** 10.2174/1573403X10666140214121152

**Published:** 2013-11

**Authors:** Georg M. Frõhlich, Richard M Lyon, Comilla Sasson, Tom Crake, Mark Whitbread, Andreas Indermuehle, Adam Timmis, Pascal Meier

**Affiliations:** 1 Cardiology, University College London Hospitals UCLH, London, UK; 2 London Air Ambulance Service HEMS;; 3 Department of Emergency, University of Colorado, Aurora, Colorado; 4 London Ambulance Service LAS; 5 Division of Cardiology, Yale Medical School, New Haven, CT; 6 Cardiology, The London Chest Hospital, London, UK

**Keywords:** Out-of-hospital cardiac arrest, resuscitation, sudden cardiac arrest, ventricular fibrillation.

## Abstract

Out-of-hospital cardiac arrest (OHCA) has attracted increasing attention over the past years because outcomes have improved impressively lately. The changes for neurological intact outcomes has been poor but several areas have achieved improving survival rates after adjusting their cardiac arrest care. The pre-hospital management is certainly key and decides whether a cardiac arrest patient can be brought back into a spontaneous circulation. However, the whole chain of resuscitation including the in-hospital care have improved also. This review describes aetiologies of OHCA, risk and potential protective factors and recent advances in the pre-hospital and in-hospital management of these patients.

## INTRODUCTION

Sudden cardiac arrest (SCA) and sudden cardiac death (SCD) refer to the sudden collapse of cardiac activity with hemodynamic compromise, either due to sustained ventricular tachycardia (VT)/ventricular fibrillation (VF), asystole or pulseless electric activity (PEA). There are also non-cardiac etiologies for sudden circulatory collapse (Table** 1**) [1]. The most common cause of cardiac arrest is acute or chronic coronary artery disease but various other cardiac and non-cardiac related diseases are also well recognised, as initially disputed (Table** 1**). Sudden out of hospital cardiac arrest accounts for around 0.5 - 1 deaths per 1000 population every year [2]. In North America alone, more than 300.000 deaths per year are attributed to SCA [3]. The survival rate has remained very low for several decades, with only 8-10 % of patients surviving to hospital discharge and in many rural areas survival is dramatically less [4]. Survival and long-term functional outcome are closely related to the underlying cause such as initial rhythm. 

## RISK FACTORS FOR SUDDEN CARDIAC DEATH

The high mortality associated with SCA emphasises the need for early identification of patients at risk. However, very little is known about risk factors. Since coronary artery disease is the most important cause of SCA, cardiovascular risk factors also increase the risk of OHCA and this is especially the case for diabetes and smoking but less so for obesity [8, 9]. 

However, our understanding of the relationship between acute myocardial ischemia and its most fatal immediateconsequence, cardiac arrhythmia, remains very limited. We know that ischemia alters repolarisation and it prolongs the QT interval [10]. The extent of QT prolongation during an acute coronary artery occlusion depends on the degree of collateralisation [10, 11]. Intriguingly, there is increasing evidence that the collateral circulation has a protective role during early ischemia. A study in 170 patients with acute anterior infarction showed lower incidence for malignant arrhythmias (defined as VF, VT or high degree atrioventricular (AV) block and lower mortality in patients with angiographically well-developed collaterals [12]. Furthermore, a well-developed collateral circulation has been associated with reduced risk for cardiac and all-cause mortality in patients with stable coronary artery disease in general [13]. 

## ELECTROPHYSIOLOGICAL RISK ASSESSMENT

Testing of the electrical vulnerability to ventricular arrhythmia remains elusive. This is primarily due to the dynamic nature of the electrophysiological behavior of the myocardium. Although invasive electrophysiological testing to evaluate the inducibility of ventricular arrhythmia has been advocated, the validity of this approach is uncertain. Non-invasive investigations have been developed to estimate the susceptibility to arrhythmias [14]. These tests examine different aspects of myocardial electrophysiology as reflected on the surface electrocardiogram (ECG), namely change of autonomic function due to increased circulating catecholamines, delay in myocardial conduction, prolongation of repolarization and stretch-induced after-depolarisations.

The clinical usefulness of these tests is controversial. The highest diagnostic yield may be a combination of tests given the limited predictive value of each individual investigation [15]. 

## THE RISK OF SCD IN ATHLETES

Overall, the risk of SCD is estimated to be between 1:50.000 and 1:300.000 in athletes over a 10-20 years period [16-18].

Due to their increased physical activity, athletes, and especially those involved in competitions, are at particular risk of SCA in the presence of conditions such as hypertrophic cardiomyopathy, abnormal origin of the coronary arteries, myocarditis, arrhythmic right ventricular cardiomyopathy (ARVC), mitral valve prolapse, aortic stenosis, coronary arteriosclerosis [19]. The European Society of Cardiology (ESC) has published guidelines for the pre-participation screening of young competitive athletes in 2005 [20]:

- Complete personal and family history and physical examination
- 12 lead ECG
- Any abnormal findings warrant further examination (e.g. echocardiography or cardiac magnetic resonance imaging (CMR)
- Reevaluation after two years

Similarly, a family screening of patients who experienced unexplained OHCA has to be considered. An ECG and stress-testing, echocardiography or magnetic resonance imaging (MRI) but also genetic testing may be useful in this setting.

## MANAGEMENT OF SCA

The European Resuscitation Council (ERC) updated their guidelines on the treatment of SCA in 2010 [21]. The main changes compared to the 2005 guidelines in Basic Life Support (BLS) were the introduction of a compression/ventilation ratio of 30:2 as compared to 15:2 to optimize maintenance of circulation and reduce the “hands-off” periods. There is increasing evidence that continuous chest compressions (“hands-only cardio- pulmonary resuscitation (CPR) without ventilation, might not entail adverse consequences on neurologic outcome, at least within the first few minutes after SCA [22].

However, the debate on the ratio of chest compressions and ventilations during CPR and the continuous adaption of guidelines may have confused lay people and prevented them from performing CPR. Nose-to-mouth or mouth-to-mouth ventilation recommended during CPR may further deter people fearing infection. Moreover, the use of automated external defibrillators (AED) is clearly recommended.

It is a matter of an ongoing debate, whether CPR should be applied immediately or whether early defibrillation should be preferred in patients after SCA. While current guidelines advocate immediate defibrillation, both approaches seem to have comparable results and in patients with a cardiac arrest >5 minutes, chest compressions before defibrillation may be superior [23]. 

Importantly, patient transfer with ongoing CPR results in reduced quality of chest compressions. However, with the new mechanical chest compression devices (e.g., LUCAS (Jolife, Lund, Sweden) and AutoPulse (Zoll Circulation, Chelmsford, Massachusetts, USA) adequate chest compression quality can be maintained during transport [24]. However, there is currently insufficient evidence for a clear advantage over manual chest compressions with regard to clinical outcomes when using the LUCAS device [25]. Similarly, the Circulation Improving Resuscitation Care (CIRC) trial assessing the effect of the AutoPulse automatic chest compression device in >4000 OHCA patients failed to show a survival benefit. However, the data of this trial are not published yet. 

The primary aim of post cardiac arrest return of spontaenous circulation (post-ROSC) care is to optimize cardio-cerebral recovery. In-hospital aims include maintaining cardiac output and cerebral perfusion, optimising systemic haemodynamics and minimising ischaemia-reperfusion injury [26]. The importance of optimal post-resuscitation care is highlighted in the recent 2010 International Liaison Committee on Resuscitation (ILCOR) Consensus on CPR Science with Treatment Recommendations (CoSTR) and ERC Guidelines [27, 28]. Table** 2** gives an overview of a recommended diagnostic work-up of patients who survived SCA.

## MANAGEMENT OF OHCA

An updated Advanced Life Support (ALS) algorithm for medical professionals is shown in Fig. (**1**). 

## ROLE OF HEART CATHETERIZATION AND PERCUTANEOUS INTERVENTION AFTER SCA

Importantly, current guidelines recommend immediate referral of patients after OHCA to a cardiac centre with onsite cardiac catheterisation facilities in patients after SCA [21]. The decision by the pre-hospital emergency medical service (EMS) provider where to admit the patient after an OHCA is crucial. Several non-randomised observational studies have demonstrated survival benefit from early angiography post-OHCA compared to no coronary angiography or percutaneous coronary intervention (PCI) [29, 30]. However, other studies have also highlighted an increased complication risk if early angiography is performed in these patients [31]. In our view, although the role of immediate coronary angiography is controversial, substantial information on the coronary circulation can guide in-hospital management beyond coronary intervention. For example, in patients with cardiogenic shock, an intra-aortic balloon pump (IABP) or other support devices (e.g. Impella 2.5) can be inserted at this occasion to augment cardiac output [32].

Moreover, approximately 80% of OHCA presenting with VF or VT are cardiac in origin and these patients may benefit from an early PCI [33]. Studies are currently under way to determine whether patients who fail to achieve return of spontaneous circulation (ROSC) at the scene and who are suspected to have obstructive CAD may benefit from PCI whilst receiving continuous CPR [34]. Importantly, PCI plays a major role in the improved survival rates [35, 36]. In a study of 714 OHCA patients referred to a tertiary centre in Paris, 435 (61%) had no obvious extracardiac cause. This subgroup underwent early coronary angiography and 70% of those had at least one significant coronary lesion [30]. 

To facilitate decision making, an ECG should be recorded as soon as possible after ROSC to assess for ST-elevation or (new) LBBB [37]. However, the ECG has a limited accuracy in the setting of SCA. The absence of ST-segment elevation does not exclude the presence of critical coronary stenoses. In approximately 50% of OHCA survivors despite the absence of ST-segment elevation in the post-arrest ECG, a significant coronary artery stenosis can be found; [29, 30] However, even though these coronary artery stenoses were regarded “significant”, it remains unclear whether these stenoses are actually the cause for the cardiac arrest and whether revascularising these lesions can improve the clinical outcome. Unfortunately, the evidence in this area is very scant, cardiac arrest patients have been excluded from most acute myocardial infarction trials, which has created a gap of evidence for these patients. While non-cardiac arrest patients with ST elevation infarctions clearly benefit from immediate angiography/ PCI, we lack data for patients after a cardiac arrest.

## HYPOTHERMIA

Based on experience with avalanche victims who had good neurological outcome despite very prolonged circulatory arrest under the circumstance of hypothermia, the concept of therapeutic hypothermia has been proposed in patients with OHCA. As to date, it is the only post-ROSC intervention shown to improve survival from OHCA [38]. Two major randomised clinical trials (the Hypothermia After Cardiac Arrest trial from Europe [39] and a smaller trial from Australia [40]) have demonstrated the efficacy of this intervention. The exact mechanism of the protective effects of mild therapeutic hypothermia (MTH) remains to be determined but probably includes a reduction of ischaemia-reperfusion injury and a reduction in oxidative stress. During cardiac arrest, brain tissue becomes ischaemic. Following ROSC, rapid re-oxygenation leads to oxygen free radical production, which can lead to secondary cell death. MTH has pleiotropic neuroprotective effects. MTH slows down cellular metabolism, altering biochemical and signaling pathways and reduces oxygen demand. Comatose (i.e., lack of meaningful response to verbal commands) adult patients with ROSC after out-of-hospital VF cardiac arrest should be cooled to 32°C to 34°C for at least 12 to 24 hours. MTH can also be considered following OHCA with non-shockable initial rhythms such as PEA or asystole but the benefit in these patients is less clear [28, 41].

Many questions surrounding MTH remain unanswered. The timing of initiation and the optimal duration of cooling are unclear. Should MTH be initiated pre-hospital or is it sufficient to start this therapy in hospital? Some experimental studies suggest that early cooling improves outcome; but a randomised clinical trial failed to show superiority for pre-hospital cooling versus in-hospital initiated cooling [42]. The time frame during which MTH can be initiated is unknown. The length of time MTH should be maintained is also uncertain. In the Australian trial patients were cooled for 12 hours [40], while in the Hypothermia After Cardiac Arrest (HACA) trial they were cooled for 24 hours [39]. Longer duration may be superior. [43]

The optimal cooling method is not yet clear [38]. External cooling methods include cold pads and cooling caps, whilst invasive cooling methods include cold intra-venous saline or intra-vascular cooling catheters. Recently, intra-nasal evaporative cooling has been shown to produce effective pre-hospital cooling [44]. However, 30 mL/kg of intravenous 4°C saline or Hartmann’s solution is the simplest and most cost effective method for pre-hospital setting. The target core temperature is 33 ± 1°C. Intravascular devices have been shown to be effective in inducing and maintaining this target temperature; however, there can be delays in inserting the intravascular catheter and this is more invasive than other techniques. Surface cooling, using cooling blankets and ice packs to the axilla, groin and neck, has also been advocated. These devices are simple, require less operator experience, and are inexpensive, but initial cooling may be slower [45]. One study has shown that both intravascular and surface cooling are equivalent in their effectiveness to reach and maintain core temperatures [46], although another showed better temperature control with an intravascular device [47]. Whichever method is used, a feedback loop is advocated to ensure target temperature compliance and to prevent overcooling. Devices with feedback control also enable better control of rewarming (usually at 0.25^o^C/h).

Despite strong evidence and clear guideline recommendations, MTH is still under-used in some regions of the world. In a recent trial of an impedance threshold device for OHCA in the United States and Canada, just 48% of the 2289 enrolled patients admitted hospital were treated with therapeutic hypothermia [48]. On the other hand, in the UK 86% of intensive care units had implemented hypothermia by 2009 [49]. Lack of resources and cost of MTH are commonly cited as barriers to implementation, despite relatively cheap options being available. 

However, in patients with MTH several side effects may occur. MTH may affect the coagulation cascade and platelet function, eventually leading to an increased bleeding risk [50, 51]. In case of bleeding, the patient should be rewarmed >35°C body temperature. Moreover, leucocyte function may be decreased resulting in an increased risk for infections [39]. On the ECG, bradycardia and a prolongation of the QT interval may be detected [52]. In rare cases, severe arrhythmias are provoked by MTH. Further, due to renal side effects, the electrolyte balance may be altered and careful monitoring is mandatory [53, 54]. Additionally, the metabolisation of several drugs may be altered by MTH [54]. 

## SECONDARY PREVENTION OF SCA

In patients who survived SCA, strategies are needed to prevent future, potentially fatal events. The mechanisms of death were evaluated in several studies. The main cause of death in these trials was low cardiac output due to progressive heart failure (45-50%), severe arrhythmia (20-35%) and non-cardiac related death (e.g. renal disease, ca. 20-30%) [55, 56]. Table** 3.** refers to the European Society of Cardiology (ESC) guidelines on decision making on implantable cardioverter defibrillator (ICD) implantation for secondary prevention of SCA due to severe arrhythmia [57, 58]. Additional therapies including VT ablation or permanent antiarrhythmic therapy may be warranted in selected cases. However, as outlined previously severe arrhythmias are closely related to an impaired left ventricular function, and an optimal heart failure management will be mandatory in this patient population [59].

## PROGNOSIS AFTER SCA

**Fig. (2):**
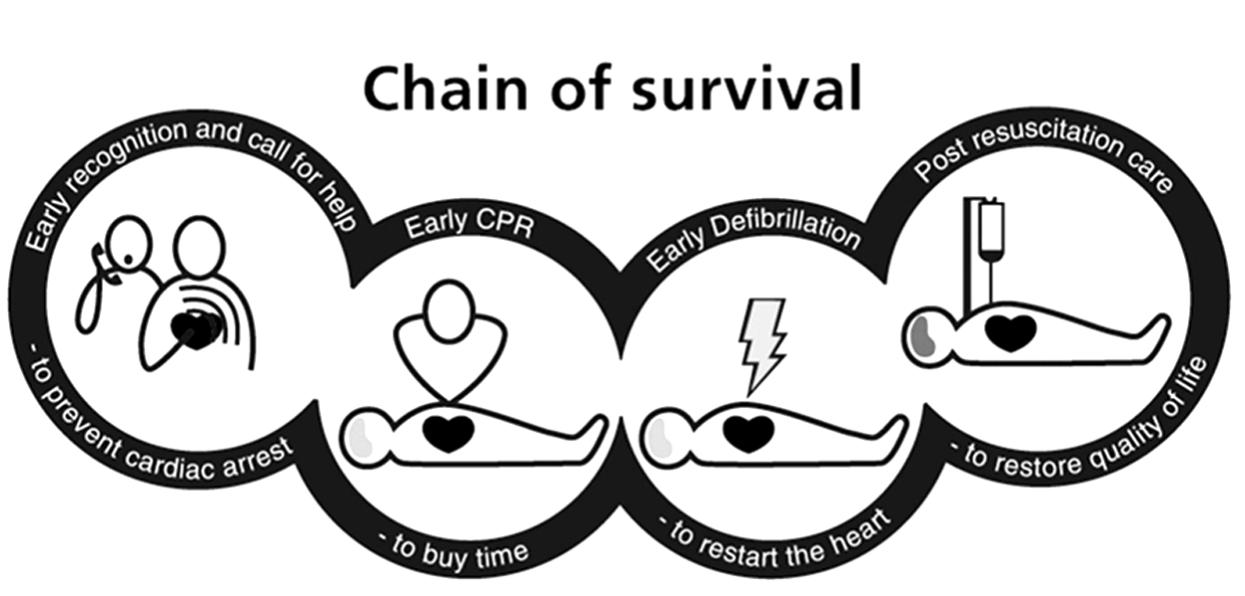
The chain of survival.

The Time to defibrillation and other factors such as bystander CPR, has not improved over precise time [60]. The chain of survival (Fig.** 2**) is changing over time and it is likely that it will soon include pre-hospital induction of hypothermia and early coronary intervention. Overall, such changes have increased the survival rate. In Sweden for instance, the survival rate after OHCA has doubled over the last years [61]. 

The prognosis after cardiac arrest is determined by several factors. In case of asystole or PEA as the initial rhythm, a prolonged cardiac arrest must be assumed and therefore only 10% of patients will survive until hospital admission [62, 63]. In contrast, outcome is much better in patients with ventricular arrhythmias, especially in those with a witnessed cardiac arrest [64]. In the vast majority of patients, ventricular fibrillation will not terminate spontaneously and the probability to survive will decline by 10% per minute of ongoing VF [65]. Ideally, a regular pulse is restored within 10 minutes of CPR [66].

In a study including 200 patients presenting with ventricular fibrillation and successful early defibrillation, 72% of patients survived until hospital admission. However, only 40% of these patients were discharged with no or mild neurologic impairments [64]. Therefore, angiography is often delayed until the neurological status of the patient can be determined accurately. Blood markers may help to estimate the impact of hypoxic brain injury, such as the levels of the protein “neurone specific enolase”, “S-100” or “IL-8”, although predictive value and accuracy varies widely in different studies [67, 68]. More precise tools to predict outcomes would help tremendously for optimal resource allocation [69]. 

## CARDIAC ARREST CENTRES

There is variation in outcome for OHCA patients depending on the hospital they are admitted to [70] and there is some evidence that mortality is lower among those admitted to intensive care units (ICUs) that treat a high volume of post-cardiac arrest patients [71]. A specialised multidisciplinary team approach to post-resuscitation care is essential. Post-resuscitation care is started by the EMS on scene and in some systems this may include pre-hospital induction of hypothermia. It is important to designate specific and clear roles to each team involved in the care and management of OHCA patients (emergency medicine, critical care, cardiology, neurology).

Trauma-centres have dramatically improved the outcomes of severely-injured patients. This model might be applicable to other medical conditions such as OHCA or stroke. Based on this premise, regionalised, coordinated resuscitation centres to care for post-OHCA patients has been proposed [72]. EMS providers should transport patients to those hospitals that are best suited for caring for OHCA victims - such hospitals would provide therapeutic hypothermia, 24/7 access to PCI facilities and availability of dedicated neurological investigations [72]. 

## CONCLUSIONS

There have been multiple recent advances in the care of OHCA patients which may have a synergistic effect. The development of cardiac arrest centres, post-OHCA management protocols, further advances in therapeutic hypothermia and primary percountaneous intervention (PPCI) in post-OHCA are likely to further improve outcomes in the future. We are moving away from the perception that survival of OHCA victims is a fortunate rare event towards a renewed sense that OHCA is often a treatable event with an increasing chance for neurologically intact survival.

## Figures and Tables

**Fig. (1):**
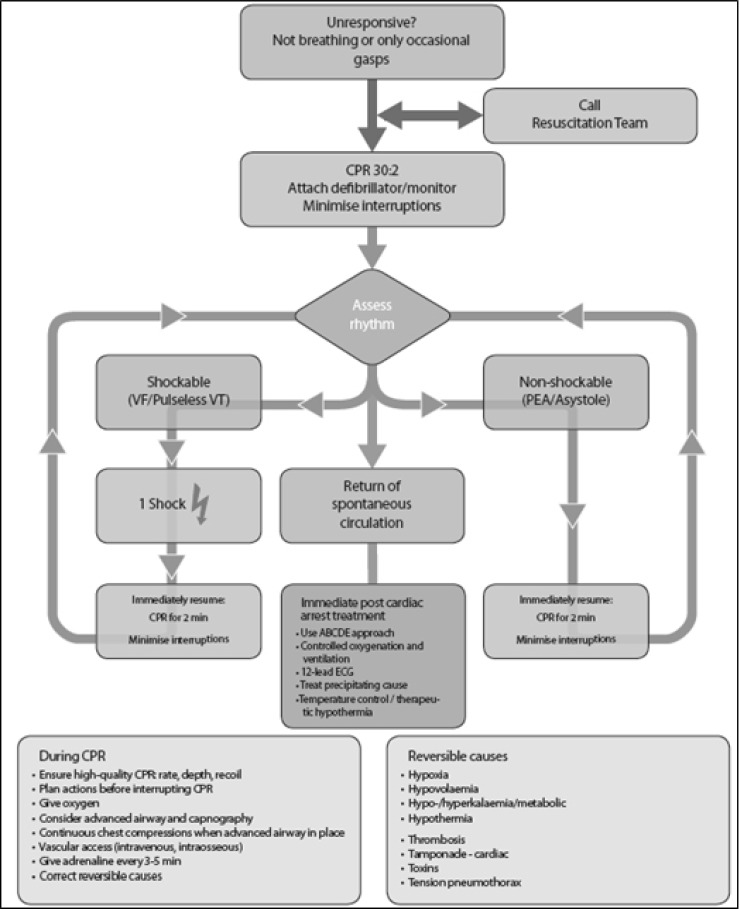
Algorithm for Advanced Life Support (ALS) [21].

**Table 1.** Causes of OHCA [5-7]

Cardiac causes	Non-cardiac causes (≈5-12%)
*Ischemia *(≈70%) coronary artery disease (CAD) heart failure not related to CAD: coronary emboli inflammatory disease vasospasm	*Electrolyte disorders* Hypo-/hyperkalemia acidosis kidney disease, dialysis
*Structural heart disease* hypertrophic cardiomyopathy dilated cardiomyopathy and heart failure arrhythmogenic right ventricular dysplasia Takotsubo cardiomyopathy myocarditis aortic dissection congenital heart disease	*Neurological illnesses* stroke subarachnoid hemorrhage *Hypoxia* *Trauma (commotio cordis)* *Massive pulmonary embolism*
*Electric disorders* Long-/short QT syndrome Wolf-Parkinson-White syndrome (WPW) Brugada syndrome idiopathic	*Drugs and drug interactions* Digoxin antiarrhythmic drugs Several antidepressant drugs *Infectious diseases and sepsis *

**Table 2.** Diagnostic Investigations for Patients After OHCA

History and physical examination Prior diagnoses of heart diseases, concomitant diseases? Family history? Medications (which could cause QT prolongation, electrolyte disorders, arrhythmia) Drug abuse Angina equivalent symptoms Signs of heart failure
Laboratory evaluation Electrolytes and renal function Blood gas including lactate and pH value, pO2 and pCO2 Serial troponin measurements, if no coronary angiography
Electrocardiogram ST-segment elevation or new left bundle branch block (LBBB) 2nd or 3rd degree heart block Signs of Brugada, ARVC, long QT, WPW, hypertrophic cardiomyopathy Pharmacologic challenge to reveal Brugada (procainamide) or polymorphic ventricular tachycardia (epinephrine)
Echocardiography Structural heart disease (hypertrophic cardiomyopathy, ARVC,...) LV-function and wall motion disturbances
Coronary angiography Confirm/exclude ischaemia as underlying condition Anomalous origin of the coronary arteries
Cardiac magnetic resonance imaging LV-function, ischemia Structural heart disease (ARVC, amyloidosis, sarcoidosis,…) Myocarditis

**Table 3.** Secondary Prevention of SCA. Decision Making on ICD Implantation

Reversible causes of OHCA *ICD not primarily recommended*	Non-reversible causes of OHCA *ICD recommended*
Acute myocardial ischemia	LVEF ≤ 35%, not in context of acute ischaemia
Electrolyte disorders in the presence /or not of antiarrhythmic drug therapy proven to be the cause of SCA	LVEF > 35% Grey area Electrophysiology studies potentially helpful Individualised decision for ICD (hypertrophic cardiomyopathy, long QT, etc.)
Arrhythmia related to acquired long QT syndrome Patient must avoid exposure to drugs	
Fulminant myocarditis	
WPW syndrome as a trigger for VF: ablation	

## ABBREVIATIONS

ROSC: = Return of Spontaneous Circulation
ALS: = Advanced Life Support
ARVC: = Arrhythmic Right Ventricular Cardiomyopathy
AED: = Automated External Defibrillators
BLS: = Basic Life Support
CPR: = Cardiopulmonary Resuscitation
ECG: = Electrocardiogram
EMS: = Emergency Medical Service
ERC: = European Resuscitation Council
ESC: = European Society of Cardiology
ICD: = Implantable Cardioverter Defibrillator
ICU: = Intensive Care Unit
IABP: = Intra-Aortic Balloon Pump
LBBB: = Left Bundle Branch Block
MRI: = Magnetic Resonance Imaging
MTH: = Mild Therapeutic Hypothermia
OHCA: = Out-of-Hospital Cardiac Arrest
PCI: = Percutaneous Coronary Intervention
PPCI: = Primary Percutaneous Coronary Intervention
PEA: = Pulseless Electric Activity
SCA: = Sudden Cardiac Arrest
SCD: = Sudden Cardiac Death
VF: = Ventricular Fibrillation
VT: = Ventricular Tachycardia
WPW: = Wolf-Parkinson-White syndrome
